# Demographic and Clinical Features of Endometrial Polyps in Patients with Endometriosis

**DOI:** 10.1155/2016/1460793

**Published:** 2016-05-03

**Authors:** Ningning Wang, Yufeng Zhang, Bin Liu

**Affiliations:** ^1^Department of Obstetrics and Gynecology, The First Affiliated Hospital, Sun Yat-sen University, No. 58th Zhongshan 2nd Road, Guangzhou 510080, China; ^2^Department of Gynecology, The Third Hospital of Nanhai, No. 45 Zhenxing Road, Lishui Town, Nanhai District, Foshan 528244, China

## Abstract

*Aims*. To compare the clinical features of endometrial polyps (EPs) between patients with endometriosis (EM) (EM group) and without EM (non-EM group).* Methods and Results*. Seventy-six cases in the EM group and 133 cases in the non-EM group underwent laparotomy or hysteroscopy and laparoscopy; later, it was confirmed that the results by pathology from July 2002 to April 2008 in the Department of Gynecology and Obstetrics at the First Affiliated Hospital of Sun Yat-sen University. The recurrence of EPs was followed up after the surgery until 2013. The following parameters were assessed: age, gravidity, parity, infertility, and menstrual cycle changes, as well as polyps diameters, locations, number, association with the revised American Fertility Society (r-AFS) classification, and their recurrence. On review, 76 EPs cases of EM group histologically resembled EPs but the majority of EPs with EM occurred in primary infertility cases and in fewer pregnancy rate women who had stable and smaller EPs without association with the AFS stage. The recurrence rate of EPs in EM group was higher than that in non-EM group.* Conclusion*. It is important to identify whether infertile patients with EM are also having EPs. Removing any coexisting EPs via hysteroscopy would be clinically helpful in treating endometriosis-related infertility in these patients.

## 1. Introduction

Endometriosis (EM) is defined as functional endometrial glands and stroma tissue that are located outside the uterine cavity. It affects approximately 2–17% of women in their reproductive years, and it typically manifests as chronic pelvic pain, congestive dysmenorrhoea, heavy menstrual bleeding, and deep dyspareunia. It is suggested that 47% of infertile women have EM [1, 2]. Although its pathogenesis is not clear, endometriosis associated with infertility is gradually accepted to be partially related to endometrial polyps (EPs) [3–6].

EPs, the local hyperplastic growth of endometrial glands and stroma covered by epithelium, can affect between 7.8% and 34.9% of women, especially infertile women [7, 8]. EPs can occur as a single polyp or multiple polyps, can be sessile or pedunculated, and can range in size from millimeters to centimeters [9, 10]. Occasionally, EPs can contain smooth muscle fibers called adenomyomatous polyps [11]. They are frequently encountered with abnormal uterine bleeding (AUB). Similar to endometriosis, EPs can also be associated with intracavitary bleeding and can present an abnormal environment for embryo implantation [12, 13]. Hysteroscopy is superior to other treatment methods because hysteroscopic polypectomy appears to improve fertility and increase pregnancy rates by using direct visualization to completely remove the polyps while leaving the adjacent endometrium intact [14–19].

In cases of infertility, EM and EPs can be closely associated with each other in some respects. For example, some studies reported a higher frequency of EPs in EM patients [3, 4, 20]. However, the characteristics of polyps in the EM patients and the manner in which they differ from those in patients without EM have not yet been elucidated. The objective of the present study was not only to evaluate the association of EM with EPs but also to investigate the characteristics of EPs in EM patients.

## 2. Patients and Methods

### 2.1. Patient Groups

A retrospective comparison was conducted of the data from 76 patients (EM group) who had been diagnosed with EM with EPs and 133 patients (non-EM group) who had been diagnosed with EPs without EM based on pathology, all of whom had undergone laparotomy or hysteroscopy and laparoscopy between July 2002 and April 2008 in the Department of Gynecology and Obstetrics at the First Affiliated Hospital of Sun Yat-sen University. The recurrence of EPs was followed up after the surgery until 2013. The two groups of patients had no internal medicine complications and had not taken any steroid hormone medications within 3 months before the surgery. There was no significant difference between the two groups with respect to the presence of uterine fibroids and adenomyosis (*P* = 0.90 and *P* = 0.67, resp.). The eutopic endometrial pathological types in the two groups were not different with respect to the menstrual cycle stage (*P* = 0.12). This study was approved by our hospital ethics committee.

### 2.2. Data Collection and Criteria

A retrospective analysis and comparison of demographic and clinical characteristics between the two groups as clinical data [age and symptoms (e.g., menstrual changes, gravida, parity, and abortion, including medical abortion and spontaneous abortion times)], operative data (EPs: size, number, location, and type; EM:r-AFS stages I–IV), and pathological data were conducted. The EPs size was measured via preoperative vaginal ultrasound and confirmed during diagnostic hysteroscopy prior to resection or by gross appearance. The sizes were estimated using the largest polyp as a reference. The cases with prolonged operative times due to multiple operations for other indications or complications were excluded from the final analysis.

The diagnosis of EPs was made by histopathological examination. Specifically, the diagnosis was made according to the presence of irregularly dilated endometrial glands and thick-walled vessels scattered within fibrotic stroma. The type of EPs was determined based on the angle between the polyp and the adjacent uterine wall. (1) Pedunculated-type polyps were defined when the angle of the polyp surface to the endometrium was <90 degrees. (2) Sessile-type polyps were those with an angle ≥90 degrees [21]. The presence and diagnostic criteria of uterine hemorrhage were divided into the following four categories [22]: (1) menorrhagia, (2) hypermenorrhea, (3) metrorrhagia, and (4) polymenorrhea. In our study, we considered hypermenorrhea together with menorrhagia as menorrhagia and metrorrhagia together with polymenorrhea as polymenorrhea because patients were often confused regarding the distinction between the different types. Histological and pathological information was also collected, including the presence and degree of the accompanying endometrium in the proliferative or secretory stage, adenomyosis, and leiomyoma, as well as the presence of EPs and EM.

### 2.3. Statistical Analysis

Statistical analysis was performed using SPSS 13 Statistical Software (SPSS Inc., Chicago, IL, USA). Multivariate logistic regression analysis was performed, in which the occurrence of EPs was used as the dependent variable, while the age, gravidity, and parity were used as independent variables. Quantitative data, such as age, polyp diameter, and number of polyps, are expressed as the means and 95% confidence intervals (95% CIs). Differences between the groups were assessed using the Mann-Whitney *U* test for continuous variables and Fisher's exact test for categorical variables. The patients' polyps, along with infertility type, menstrual cycle changes, polyp location, and EM r-AFS stage, were compared with the Pearson chi-square test and Fisher's exact test for qualitative variables. A *P* value < 0.05 was considered to be statistically significant.

## 3. Results

The average age of the EPs patients in the EM group was 38.37 ± 0.74 years, while that of the EPs in non-EM group was 40.0 ± 10.55 years (*P* > 0.05). After a follow-up period to 2013, 76 patients of EM and 133 patients of non-EM group were contacted for a phone interview. Two persons could not be reached by the phone.

### 3.1. Endometrial Polyps in Patients with Endometriosis Related to Decrease in Number of Pregnancies

The incidence of EPs in EM group was not related to the age and gravidity. However, it was negatively related to parity (*P* = 0, OR = 0.41); specifically, the less the parity, the higher the incidence of EPs in EM group (Table 1). The incidence of infertility in EPs patients of the EM group was significantly higher than that in EPs patients of non-EM group (*P* = 0.00), and the incidence of EPs in EM patients was significantly increased in primary infertility patients (Table 2).

### 3.2. Clinical and Pathological Features of Endometriosis Patients with Endometrial Polyps

EPs with EM tended to be a stationary state; that is, the original menstrual cycle and menstrual volume were maintained. However, EPs in non-EM group occurred with menorrhagia (*P* = 0.00) (Table 3). Further the EPs size and the menstrual changes in the two groups are not related; that is, the size of the EM patients with EPs has no effect on menstruation change comparing between menstrual cycles (*F* = 2.02, *P* = 0.14) and between the two groups in the same menstrual cycle (*F* = 2.08, *P* = 0.15) (Table 4). There was no difference when we compared the relationship between the types of menstruation and the locations of the EPs in the two groups. But the polyps in the corpus in the two groups were closely related to the menorrhagia status (Table 5). The EPs in patients with EM were smaller (Table 6), concentrated in the corpus and uterus, and had a sessile trend. There was no significant difference with respect to the number, distribution, and type of polyps between the two groups (Table 7). The size and number of EPs in patients in EM group were not correlated with the r-AFS stage (*P* = 0.19, *F* = 1.64 and *P* = 0.88, *F* = 0.22, resp.) (Table 8).

### 3.3. The Recurrence of Endometrial Polyps in Two Groups

The recurrence rate of EPs in EM patients in our study was higher in EM patients with EPs, and there was a positive association between the recurrence rate and follow-up period; specifically, the 2-year recurrence rate was 23.08% postoperatively, whereas the 5-year recurrence rate was as high as 56.41% (Table 9).

## 4. Discussion

The precise pathogenesis of endometriosis in patients with EPs is not clear. However, the most widely accepted characteristic mechanism for endometriosis is retrograde menstruation with the transport of endometrial cells, metaplasia of coelomic epithelium, and hematogenous or lymphatic spread of endometrial cells. Other factors, such as genetic, immunological, and inflammatory factors, are involved in this process, in which eutopic endometrial fragments become implanted in the pelvis or other organs. A combination of these theories is likely to characterize the features of endometriosis. Furthermore, because blood reflux is common when women are of reproductive age, the eutopic endometrial status in endometriosis patients is mainly considered to be abnormal at the same time [23–25].

Previous studies [17, 18, 20], together with our research, revealed a significantly increased risk of EPs in women with endometriosis compared with those without endometriosis. Moreover, Zheng et al. [20] indicated that endometriosis patients have a significantly higher risk of EPs, especially patients with endometriosis greater than stage I. Our research further reported that EPs associated with endometriosis exhibited the same structure as other polyps and often occurred in infertile women, especially in those with primary infertility, or there were fewer pregnancies in women with endometriosis. Based on our present study, EMs with EPs are not closely related to the clinical symptoms and r-AFS stage.

Furthermore, our results showed that the EPs associated with EM were in a relatively quiescent state; that is, there is little risk of the severity of menstrual disorders increasing, and the original menstruation pattern is often maintained. It is recommended for EM patients with infertility to have routine vaginal ultrasound and hysteroscopy examinations to assess EPs. At the same time, this examination should be conducted more carefully because polyps associated with EM are generally distributed in a similar manner with other polyps but have a smaller size. The recurrence rate of EPs in EM patients in our study was higher in EM patients with EPs, and there was a positive association between the recurrence rate and follow-up period; specifically, the 2-year recurrence rate was 23.08% postoperatively, whereas the 5-year recurrence rate was as high as 56.41%.

There could be intrinsic factors that make EM more likely to occur in association with EPs, which should mainly be associated with infertility. Some authors found both EM and EPs that exhibited an overgrowth of the endometrium, a process that requires the support of estrogen. Additionally, previous research revealed that the expression patterns of estrogen receptor (ER) and aromatase are both altered in EM and EPs patients [26–28]. Additionally, increased proliferation and decreased apoptosis have been observed in the eutopic endometrium in patients with EM [24, 25]. An altered estrogen metabolism with increased proliferation and decreased apoptosis in the eutopic endometrium of women with EM could facilitate the formation of EPs.

EPs formed when the local hormone and its receptor were abnormal. At the same time, it is possible that the vessel axis of the functional polyps could actually originate from the evolution of the vascular changes that are associated with endometritis. Inflammatory factors could play an important role in EPs formation in association with EM-related infertility. In the eutopic endometrium of EM patients, vascular endothelial growth factor (VEGF), matrix metalloproteinases (MMP) 1, 2, and 9, and angiogenesis factors 1 and 2 levels were higher than those in normal endometrium [29–31]. Thus, the vascular growth factor associated with VEGF-A expression may coexist differently than in non-EM patient. If there is a difference in the VEGF-A levels between EM patients with EPs with and without primary infertility, the EPs in patients with primary infertility must be studied. Additionally, it is worthwhile to determine whether local estrogen and its receptors that are associated with inflammatory factor regulation affect the formation of EPs in EM patients with infertility.

Based on the clinical analysis, patients with EM combined with EPs have smaller polyps size, exhibiting unchanged menstrual cycle and higher recurrence rate and having high rates of primary infertility or fewer pregnancies in patient complaints. It is important to identify whether infertile patients with EM are also having EPs. Hysteroscopic polypectomy together with the removal of endometriotic foci will significantly increase the likelihood of achieving a pregnancy.

## Figures and Tables

**Table 1:** Age, gravida, and parity related to endometrial polyps associated with endometriosis.

Independent variable	*B*	*χ* ^2^	*P*	OR
Constant	0.32	0.17	0.68	1.38
Age	−0.01	0.20	0.66	0.99
Gravida	−0.01	0.00	0.93	0.99
Parity	−0.90	9.14	0.00	0.41

**Table 2:** Endometrial polyps associated with endometriosis and infertility.

Group	*n*	Primary infertility (%)	Secondary infertility (%)	Pregnancy (%)	*χ* ^2^	*P*
EM	76	27 (35.5)	7 (9.2)	42 (55.3)	32.06	0.00
Non-EM	133	8 (6.0)	9 (6.8)	116 (87.2)		

**Table 3:** Comparison of the menstrual cycles between the two groups.

	EMs (%)	Non-EMs (%)	*χ* ^2^	*P*
Menorrhagia	23 (30.3)	78 (58.6)	16.23	0.00
Polymenorrhea	12 (15.8)	16 (12.1)		
Unchanged	41 (53.9)	39 (29.3)		

**Table 4:** Relationship between the menstrual cycle and the size of the endometrial polyps in the two groups (mm, mean ± SD).

Groups	Menstrual cycle	*n*	EP diameter (mm) ($\overset{-}{x}\pm s$)
EM	Menorrhagia	23	11.22 ± 8.10
	Polymenorrhea	12	9.91 ± 10.10
	Unchanged	41	8.29 ± 7.28
Non-EM	Menorrhagia	78	13.62 ± 12.38
	Polymenorrhea	16	10.50 ± 9.14
	Unchanged	39	10.72 ± 7.57

**Table 5:** The association of the endometrial polyp locations with menstruation in the two groups.

Menstruation	Group	*n*	EPs location				*χ* ^2^	*P*
			Corpus	Horn	Cervix	Fundus		
Menorrhagia	EM	23	15	3	2	3	2.06	0.73
	Non-EM	78	56	12	6	4		
Polymenorrhea	EM	12	12	0	0	0	2.52	0.28
	Non-EM	16	13	0	1	2		
Unchanged	EM	41	34	4	1	2	1.31	0.86
	Non-EM	39	31	5	1	2		

**Table 6:** Comparison of the sizes of the polyps in two groups (mm, mean ± SD).

Group	*N*	Polyp diameter (mm) ($\overset{-}{x}\pm s$)	*t*	*P*
EM	76	9.43 ± 8.01	−2.08	0.04
Non-EM	133	12.39 ± 10.84		

**Table 7:** Comparison of the characteristics of the polyps in two groups.

	EM	Non-EM	*χ* ^2^	*P*
Number				
1	58	95	1.44	0.70
≥2	18	38		
Location				
Corpus	61	100	2.37	0.67
Horn	7	17		
Cervix	3	8		
Fundus	5	8		
Type				
Sessile	4	2	2.45	0.19
Pedunculated	72	131		

**Table 8:** Endometrial polyps in endometriosis patients associated with different r-AFS stages.

r-AFS stage	*n*	Diameter of the polyps (mm) ($\overset{-}{x}\pm s$)	Number of polyps
Stage I	31	9.10 ± 7.93	1.81 ± 2.40
Stage II	10	5.10 ± 4.43	1.90 ± 2.51
Stage III	23	10.26 ± 8.25	2.35 ± 2.71
Stage IV	12	12.33 ± 9.28	2.02 ± 2.31

**Table 9:** The recurrence of endometrial polyps in the two groups.

Follow-up	EM (%) 39/76	Non-EM (%) 40/133	*χ* ^2^	*P*
≤2 yr	9 (23.08)	8 (20.00)	16.23	0.00
2–5 yr	13 (33.33)	11 (27.50)		
≥5 yr	17 (43.59)	21 (52.50)		
